# The Role of the Olfactory System in Obesity and Metabolism in Humans: A Systematic Review and Meta-Analysis

**DOI:** 10.3390/metabo14010016

**Published:** 2023-12-25

**Authors:** Lolita Matiashova, Anouk Lisa Hoogkamer, Katharina Timper

**Affiliations:** 1 Clinic of Endocrinology, Diabetes and Metabolism, University Hospital Basel, 4031 Basel, Switzerland; anouklisa.hoogkamer@usb.ch (A.L.H.); or katharina.timper@usb.ch (K.T.); 2 Department of Biomedicine, University of Basel, 4031 Basel, Switzerland

**Keywords:** olfactory function, metabolism, obesity, smell, odor, weight management, bariatric surgery outcomes, genetic variations in olfactory receptors, metabolic syndrome

## Abstract

Obesity, linked to chronic diseases, poses a global health challenge. While the role of the olfactory system in energy homeostasis is well-documented in rodents, its role in metabolism regulation and obesity in humans remains understudied. This review examines the interplay between olfactory function and metabolic alterations in human obesity and the effects of bariatric surgery on olfactory capabilities in humans. Adhering to PRISMA guidelines, a systematic review and meta-analysis was conducted, focusing exclusively on original human studies. From 51 articles, 14 were selected for the meta-analysis. It was found that variations in olfactory receptor genes influence the susceptibility to odors and predisposition to weight gain and poor eating habits. Bariatric surgery, particularly sleeve gastrectomy, shows significant improvements in olfactory function (SMD 2.37, 95% CI [0.96, 3.77], I = 92%, *p* = 0.001), especially regarding the olfactory threshold (SMD −1.65, 95% CI [−3.03, −0.27], I = 81%, *p* = 0.02). There is a bidirectional relationship between olfactory function and metabolism in humans. Bariatric surgery improves olfactory perception in obese patients, but it is still unclear if impacting the olfactory system directly affects eating behavior and the energy balance. However, these findings open novel avenues for future studies addressing the olfactory system as a novel target to alter systemic metabolism in humans.

## 1. Introduction

Obesity, resulting from a dysregulated balance between caloric intake and energy expenditure [1], represents a global socioeconomic health burden with epidemic dimensions worldwide [2,3,4]. Obesity is a major risk factor for chronic non-communicable diseases (NCDs) such as cardiovascular diseases, type 2 diabetes, fatty liver disease, and cancer [5], contributing to increased disease burden, mortality, and healthcare costs [6]. 

The brain, particularly the hypothalamus, is a master regulator of whole-body energy homeostasis [1]. Specialized neurons within the hypothalamus, such as anorexigenic proopiomelanocortin (POMC)- and orexigenic agouti-related peptide (AgRP)/neuropeptide Y (NPY)-expressing neurons, sense the nutritional status of the organism and integrate this information into a coordinated feedback regulation of food intake, glucose, and energy homeostasis [1,7]. However, metabolic regulation involves numerous interconnected systems beyond the hypothalamus, encompassing not only various other brain regions but other aspects such as digestive enzymes secretion, insulin release, and psychological factors influencing eating behavior [8].

The role of olfaction extends beyond mere sensory perception, deeply intertwining with hypothalamic function. Hunger enhances olfactory acuity, while satiety reduces it, indicating a reciprocal relationship between food intake and olfactory sensitivity [9,10,11,12]. Odors sensed by the olfactory system change the activation status of hypothalamic AgRP and POMC neurons, thereby modulating appetite and satiety circuits [13,14,15]. Furthermore, olfactory cues also influence thermogenesis and peripheral metabolism, possibly mediated by olfactomedin 2 (OLFM2), a protein that plays a significant role in energy balance modulation [16,17,18]. The integration of olfactory signals with hypothalamic regulation underscores a complex but crucial aspect of the energy balance, influencing feeding behavior, thermogenesis, and overall systemic metabolism. Understanding this intricate network opens new avenues for treatment strategies for overweight and obese individuals, as well as associated metabolic diseases, focusing on the sensory perception of food and its metabolic consequences [15,19].

Olfactory receptors (ORs), part of the G protein-coupled receptor family, play a crucial role in olfactory signal transduction. The binding of odorants leads to the activation of ORs and thus the G protein (Golf), resulting in increased intracellular cyclic AMP levels and neuronal depolarization [20,21]. The vast array of ORs, each detecting specific odorants, along with their combinatorial coding, underscores the complexity of olfactory perception. This molecular framework paves the way for hypothesizing potential alterations in human olfactory mechanisms, especially in disorders like obesity, where changes in olfactory perception are observed but not molecularly delineated [22].

Additionally, odor binding proteins (OBPs) in the nasal mucus, particularly OBPII2, play an important role in facilitating the interaction of odorants with ORs. The rs2590498 polymorphism in the OBPIIa gene, a specific variant of OBPII2, has been associated with variations in the olfactory threshold and the intensity of perceived odors in healthy individuals [23].

Growing evidence suggests that olfactory function, or the sense of smell, is altered in the obesity state. Specifically, olfactory performance seems to diminish linearly with an increase in body mass index (BMI) [24]. Patel et al. observed that 80% of people with obesity reported a decrease in olfactory function [2,4]. A recent meta-analysis reported an impaired odor threshold with increasing body mass index (BMI), while odor discrimination and sensitivity were not changed [25,26,27,28,29,30,31,32,33]. 

An altered olfactory performance might impact food choices, potentially fostering overeating and obesity in a modern food environment. Along this line, a study demonstrated an increased sensitivity to odors of energy-dense food in individuals with obesity, suggesting that the brain might amplify autonomic responses to high-rewarding food [34,35,36]. Furthermore, the concept of a sensory-specific appetite is worth mentioning, where the scent of specific foods increases the appetite for and the consumption of that particular food product, and even of other distinct food items [37,38,39]. For instance, exposure to the smell of a banana increases the desire to ingest a banana as well as the desire to eat chocolate and brownies [39,40]. Along the same line, exposure to the odor of pears before lunch leads to a preference for a fruit dessert [41]. It has been suggested that odors may contain information about nutrients and be perceived even before food ingestion [42]. Additionally, recent research highlights how factors such as odor exposure time and concentration can directly influence food intake in humans. In a study with healthy female participants, prolonged exposure (18 s) to a high concentration of tomato soup aroma resulted in a 9% reduction in food intake compared to shorter exposure times and lower concentrations. This suggests that manipulating the retronasal aroma release can significantly affect eating behavior, underlining the potential of olfactory cues in regulating satiety and food consumption [43]. Complementing these findings, another study revealed that the satiating effect of a beverage can be enhanced when its retronasal aroma release mimics that of solid foods, indicating the significant role of aroma profiles in influencing satiety [44].

**Figure 1:**
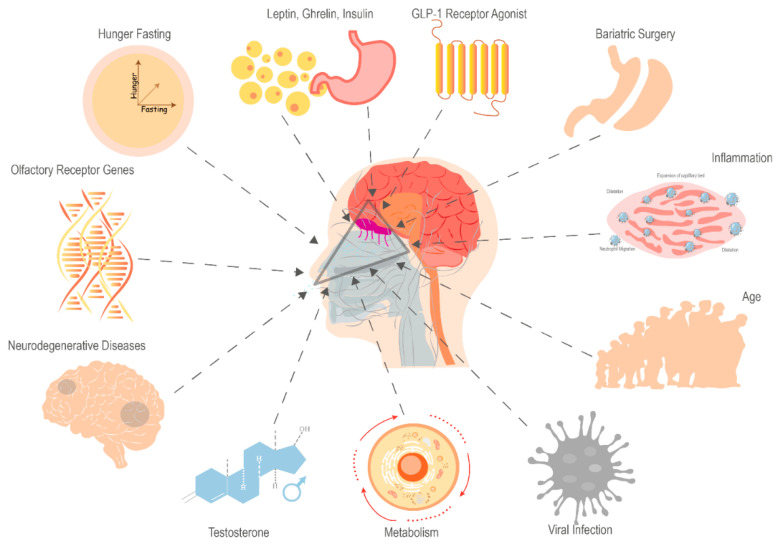
Factors Impacting Olfactory Sensory Perception (Created using Adobe Illustrator).

In rodents, the visual and olfactory sensing of food, without any nutrient ingestion, is sufficient to reverse the effects of fasting on hypothalamic neuron activity [45,46,47]. This highlights the complex interplay between olfaction and metabolic processes, extending beyond the hypothalamus to digestive enzymes secretion, insulin release, and psychological influences on eating behavior [9]. Therefore, olfactory sensing-dependent changes in hypothalamic neuron activity may be influenced by a variety of variables, such as the feeding state [48], hormones [49], age [50], respiratory infection [51], and neurodegenerative diseases [52] (Figure 1).

Additionally, environmental and social factors, including cultural norms, food availability, and social settings, play a significant role in modulating eating behavior and metabolic outcomes in interaction with olfactory perception [53]. Genetic and epigenetic variations also impact how odors are perceived and influence eating behavior and metabolism [19]. The burgeoning research on the gut–brain axis and gut microbiota further underscores the complexity of these interactions in metabolic regulation [18].

Alterations in food preference and taste are widely reported after bariatric surgery (BS) [54,55]. BS is the gold standard for the treatment of morbid obesity as it is an efficient [56] and safe method [57] to reduce the BMI and associated comorbidities in the long-term [58], leading to an overall profound reduction in cardiovascular risk [59] and an increase in life expectancy [60]. One study reported that almost 97% of people who underwent bariatric surgery experienced changes in food-related taste, smell, or preferences [61,62]. However, only a few studies investigated the influence of BS on olfactory function. While some studies reported an overall improvement in olfactory function upon BS, others found effects depending on the type of BS or no effect at all [63]. However, also in regard to olfactory changes upon BS, the methods used to assess olfactory function varied significantly between the studies and ranged from self-reported questionnaires to validated olfactory function test batteries [56].

Human research in this area is critical due to notable differences in the olfactory system and eating behavior between humans and animals. For example, humans have a more complex relationship with food that encompasses cultural, emotional, and social dimensions, contrary to most laboratory animals. Additionally, the human olfactory system, while having fewer receptors than most animals, is able to process a vast array of complex odors, influencing dietary choices in a way that is not observed in animal models [53].

The objective of this systematic review and meta-analysis is to investigate the relationship between olfactory function and obesity, including associated metabolic alterations in humans, and to examine the effects of bariatric surgery on olfactory function. The analysis will differentiate outcomes based on the type of surgical procedure and the specific tools used for olfactory function assessment.

## 2. Materials and Methods

The protocol for this systematic review and meta-analysis was registered on PROSPERO (CRD42022355091), an international database of systematic reviews and meta-analyses. The literature review and reporting were conducted according to the Preferred Reporting Items for Systematic reviews and Meta-Analyses (PRISMA). The ROBINS-E instrument was used for non-randomized trials to define risk of bias [57]. The Rayyan.ai research collaboration platform was used by two independent reviewers to include studies in a systematic review in a blinded manner [64]. The Review Manager (RevMan) version 5.4 of the Cochrane Collaboration, 2020, was used for meta-analysis [65]. 

### 2.1. Source and Methods of Data Retrieval 

The electronic databases PubMed, Web of Science, and Scopus were searched for eligible studies without publication time limits on 1 August 2022. The language of original studies was restricted to English. The following keywords were used to search the databases: olfactory function OR smell OR odor OR odour OR hyposmia AND obesity OR extra weight OR weight OR metabolic disease OR metabolic function (detailed search strategy can be found in PROSPERO CRD42022355091). 

Inclusion Criteria: The scope of this systematic review and meta-analysis was limited to original research studies involving human subjects, with no restrictions on age. The range of eligible study designs included randomized and non-randomized trials, as well as observational, cohort, and cross-sectional studies. Additionally, we established a defined population as a prerequisite for eligibility to ensure consistency and comparability across studies.

Exclusion Criteria: Studies were excluded from the analysis if they involved subjects with acute infections, including acute respiratory infection and COVID-19, or neoplastic diseases. Animal studies were not considered. Non-original studies, case reports, study protocols, and letters to editors were also deemed ineligible for inclusion. 

### 2.2. Data Extraction

**Figure 2:**
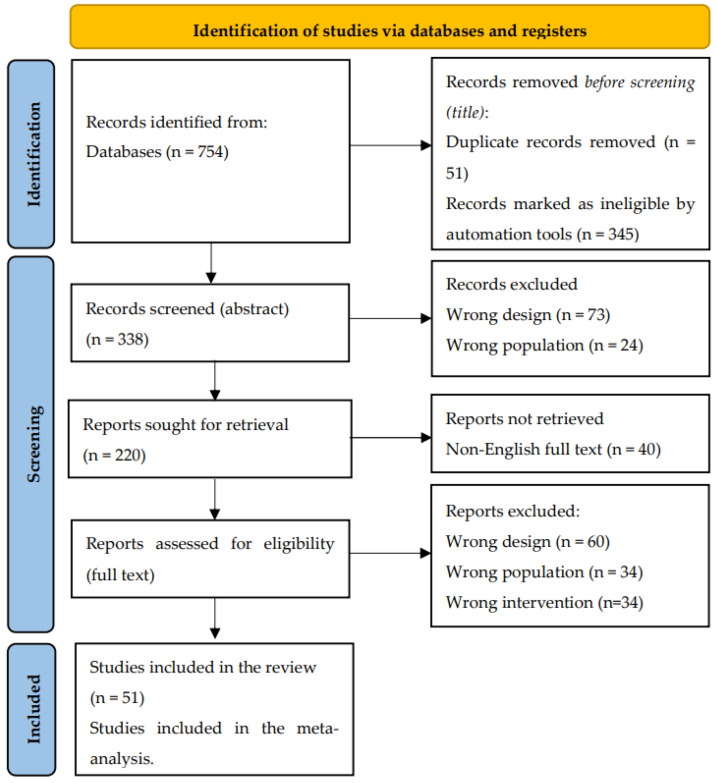
Adapted from PRISMA Flow-Chart 2020 [66].

Two independent researchers conducted a comprehensive literature search. The identified studies were subsequently uploaded onto the Rayyan.ai software, designed to streamline the process of blind inclusion and exclusion by two independent reviewers. To ensure thorough data extraction, the reference lists of all included studies were manually examined for additional potential sources. Any discrepancies in the selection process were mitigated through discussion and, if necessary, a third senior author was consulted. The entire search and selection process was systematically documented using a PRISMA flow-chart (Figure 2) [66].

Following the selection of eligible studies, pertinent information was collated using the Rayyan.ai software. Details such as the lead author, publication year, country of origin, study design, population demographics, and reported outcomes were recorded. Additionally, specific information regarding the intervention’s content and components was analyzed. If required, information about certain intervention characteristics was sourced from earlier publications, as identified from the reference list of the original article.

When authors employed multiple instruments to measure identical outcomes, the data were primarily extracted from the most relevant instrument, as decided by consensus after scrutinizing the wording of each item. A parallel procedure was followed when multiple subscales of instruments were reported instead of global scores.

The quality and potential bias of each study were evaluated using the Cochrane ROBINS-E instrument [57]. This entailed assessing various factors such as selection bias, performance bias, detection bias, attrition bias, and reporting bias, among others. Each study was consequently categorized as having “low”, “high”, or “unclear” levels of bias.

### 2.3. Meta-Analysis

Data from bariatric surgery studies were processed using the Review Manager 5.4 software. We calculated the mean difference in olfactory function before and after intervention for each study, accompanied by a 95% confidence interval (CI), which was then juxtaposed with the control group. Data were stratified based on the olfactory function test employed (sniffin’ sticks test with threshold, discrimination and identification (TDI) scoring, visual analogue scale (VAS)) and the specific type of bariatric surgery (Sleeve Gastrectomy (SG) and Roux-en-Y Gastric Bypass (RYGB)).

For dichotomous variables, odds ratios were determined using the Mantel–Haenszel test, with a random-effects model for the analysis, and results were presented with a 95% confidence interval. Conversely, for continuous data, the hazard ratio was computed via the inverse variance method, applying a fixed-effects model, and using the standard mean deviation and effect size as the measure of effect. The choice of this measure was necessitated by the diverse tests used across studies, warranting standardization for effective comparison. Computed hazard ratios were similarly represented with a 95% confidence interval. Due to insufficient data, a subgroup analysis was not carried out.

The heterogeneity among studies was evaluated with a Chi-squared (χ²) test, and the overall effect of the interventions was calculated using the Z-test. This meta-analytic statistical approach aimed to deliver a thorough and standardized comparison of diverse olfactory function outcomes upon different bariatric surgical procedures. 

Lastly, effect sizes were calculated based on the outcome data from the experimental and control groups of each study, facilitating a comprehensive comparison and analysis.

## 3. Results

The systematic identification and screening of electronic databases, following the methods described above, yielded 51 articles meeting the eligibility criteria. This systematic review includes studies which highlight (1) the connection between olfactory genes and their impact on olfactory function; and (2) the impact of metabolism on olfactory function; (3) the impact of obesity on olfactory function. The meta-analysis was limited to studies presenting quantitative evidence about the effect of bariatric surgery on olfactory function. Only these studies provided comparable quantitative data from olfactory function tests such as the “Sniffin’ Sticks” test and the VAS. 

The most frequent form of bias identified across all articles was selection bias, which occurs when a participant’s eligibility for bariatric surgery is determined based on his/her condition, in accordance with specific guidelines. Performance bias was another common bias type, which emerged when a limited number of surgeons performed the surgery, introducing potential bias due to variation in surgeons’ skills and techniques. Many articles lacked enough information to accurately assess the risk of bias.

### 3.1. Variations in Olfactory Receptor Genes and Metabolism

The olfaction phenotype is determined based on variations in olfactory receptor genes, encoding a large number of olfactory receptors (ORs) [67]. We found 18 original studies reporting an association between variations in OR genes and systemic metabolism.

Currently, variations in 23 known OR genes are associated with changes in metabolism, body weight, visceral fat, and eating behavior (Table 1). In the Quebec Family Study, sequencing of the *OR7D4* gene revealed seven single nucleotide polymorphisms (SNPs) associated with body weight and eating behavior. Specifically, rs2878329 and rs8109935 were associated with BMI, body fat percentage, waist circumference, susceptibility to hunger, and dietary restriction capacity [68]. The SNP of rs2878329 was also associated with the severity of adiposity, hunger, and conscious changes in dietary behavior. Both positive and negative associations with the amount of abdominal fat were observed in seven other *OR7D4* SNPs. Additionally, the authors investigated six other OR genes; among them, *OR7G3* exhibited a positive association with altered eating behavior and fat tissue mass. Specifically, the SNP rs10414255 of the *M29V* OR was related to increased hunger, BMI, cognitive dietary restraint, and percentage of body fat [68].

Furthermore, the Methyl Epigenome Network Association (MENA) project demonstrated the association of BMI and waist circumference (WC) with 15 CpG sites of olfactory pathway genes, four of which are OR genes. The analysis revealed that the methylation pattern of the *OR4D2* and *OR2Y1* genes was correlated with daily total energy and macronutrient intake [69].

In a genome-wide association study (GWAS), variants of three olfactory genes, *OR4P4*, *OR4S2* and *OR4C6*, were found to be associated with obesity [70].

Conversely, specific diets have shown significant impact on olfactory signaling pathways, primarily through the modulation of distinct olfactory receptor (OR) genes. In the study by Vink et al. [71] (for analysis plasma was used), a very-low-calorie diet (VLCD) was associated with a substantial downregulation of various OR genes. Notably, this dietary intervention led to the differential expression of a significant number of genes (6135 in the VLCD group), including those involved in metabolism, mitochondrial functioning, and olfactory regulation. Particularly, gene sets related to oxidative phosphorylation, lipid metabolism, and olfactory signaling were found to be downregulated in the VLCD group compared to a low-calorie diet (LCD) group.

Furthermore, a genome-wide association study [72] explored the effects of protein quantity in diets during energy restriction on OR gene expression in white adipose tissue (WAT). This study revealed that high-protein energy restriction (HP-ER) diets resulted in distinct gene expression changes compared to normal-protein energy restriction (NP-ER) diets. A total of 1869 genes showed significant expression changes in the HP-ER group. Notably, upon HP-ER diets, gene sets involved in cell cycle upregulation, G protein-coupled receptor (GPCR) signaling, olfactory, and nitrogen metabolism-related pathways were observed. In contrast, the NP-ER diet led to a downregulation of pathways related to the inflammasome, adaptive immune response, immune cell infiltration, and cell cycle.

Overall, these findings underscore the complex interplay between dietary composition and genetic regulation in the context of olfactory signaling pathways. They also highlight the potential of dietary interventions in modulating gene expression related to olfaction, metabolism, and immune responses.

Genetic variations in OR genes may influence olfactory sensing, which in turn can drive food preferences, potentially contributing to obesity [40,73]. For instance, genetic variations in the *OR7D4* gene not only relate to the ability to detect androsterone in cooked pork but are also linked to measures of total, visceral, and subcutaneous adipose tissue mass [74,75] (Table 2). In Table 3, an overview of variants in OR genes and related preferences for certain odors is presented.

In a study conducted by Ortega et al., an intriguing observation was made regarding olfactory sensitivity in non-smoking women. The study found that women with the AVI/AVI haplotypes of the *TAS2R38* gene showed a negative correlation with olfactory odor sensitivity, marked by a decrease of 8.6% (*p* = 0.03). This finding was part of a broader investigation into the genetic variations of the TAS2R38 bitter taste receptor, which has revealed significant associations with obesity. These genetic variations are seen to influence not only taste perception but also phenotypic and clinical outcomes related to extreme weight conditions. The role of *TAS2R38* variations extends beyond taste, affecting nutrient sensing and energy metabolism, and potentially impacting olfactory capacity and immune traits. However, it is important to note that the relationship between these *TAS2R38* variants and BMI, particularly in the context of obesity, anorexia, or normal body weight, necessitates more in-depth research to unravel the underlying mechanisms. Overall, these findings highlight the complex interplay between genetics, sensory perception, and metabolic health, opening new avenues for the understanding of obesity and related conditions [76]. These findings were corroborated by a comprehensive review [25], which analyzed data from multiple studies assessing the olfactory function of individuals with different body weights. The review revealed that obesity is often associated with diminished olfactory detection and discrimination abilities. Interestingly, some studies observed an increase in odor sensitivity in individuals with a higher BMI. Moreover, bariatric surgery, particularly sleeve gastrectomy, was found to improve olfactory functions, suggesting a direct link between obesity-related metabolic changes and olfactory perception. These observations, coupled with the findings of another study [77], indicate a complex interaction between body weight, metabolic health, and olfactory sensory perception, further elucidating the intricate relationship between obesity and alterations in taste and smell perception [25,77]. 

**Table 1:** Trials investigating variations in olfactory genes and their association with metabolic parameters.

Study and Number of Participants (N) Age y.o. BMI kg/m^2^	Method	Chromosome Gene Variations SNPs	Positive Association	Negative Association
Choquette [68] 2012 N = 890 Age = 43.7 ± 16.8 y.o. BMI = 27.9 ± 7.6 kg/m^2^	Direct sequencing in the Quebec Family Study	19p13 *OR7D4* c.-466	No association	
		19p13 *OR7D4* rs56139543	VAT	
		19p13 *OR7D4* rs1235784	TAT, SAT	
		19p13 *OR7D4* rs10421711	Hunger	
		19p13 *OR7D4* rs2878329		BMI, WC, BF, hunger, restraint
		19p13 *OR7D4* rs61729907	VAT	
		19p13 *OR7D4* rs5020278		
		19p13 *OR7D4* rs8109935		Restraint, hunger, BMI, BF
		19p13 *OR7D4* rs61732676	No association	
		19p13 *OR7G1* rs7246980		VAT
		19p13 *OR7G3* rs10414255	Hunger, BMI, BF	Restraint,
		19p13 *OR7E24* rs2240927		Disinhibition
Jarick [70] 2010 N = 453 N = 435 control	Genotyping by the Affymetrix Genome-Wide Human SNP Array 6.0 (PCR)	11q11 *OR4P4* rs9804659	Obesity	
		11q11 *OR4S2*		
		11q11 *OR4C6*		
Ortega [76] 2016 N = 210 women BMI = 34 ± 12 kg/m^2^ N 52 = 16.5 ± 1.3 y.o., N 86 = 21.5 ± 2.8 y.o., N 72 = 41.1 ± 7.7 y.o.	Genotyped by means of allelic discrimination assays, using a LightCyclerR	TAS2R38 AVI/AVI haplotypes arraying	Non-smoker women showed decreased smelling sensitivity. No connection to BMI	
Ramos-Lopez [69] 2019 N = 474 Age = 47.2 ± 14.1 y.o. BMI = 30.1 ± 5.6 kg/m^2^	Nutriepigenomic analysis from Methyl Epigenome Network Association	*OR4D2* cg02874396	BMI, WC, daily intakes of total energy, carbohydrates, protein, fat	
		*OR51A7* cg00467296	BMI, WC	
		*OR2T34* cg13441213		
		*OR2Y1* cg18482656	BMI, WC, daily intakes of total energy, carbohydrates, protein, fat	
		*SLC8A1* cg19302979	BMI, WC	
		*SLC8A1* cg12498094		
		*ANO2* cg10610428		
		*PDE2A* cg07736155		
		*CALML3* cg17283169		
		*GNG7* cg02849894		
		*CALML6* cg15102821		
		*CALML6* cg15819352		
		*PRKG1* cg16401207		
		*PRKG1* cg24609819		
		*CAMK2D* cg13801347		
Sun [78] 2022 N= 301 N = 307 control Age = 53.51 ± 11.1 y.o. Age = 51.20 ± 14.5 y.o. control	Bio Miao Biological Technology (PCR)	17 OR4D1 rs8071251 rs7218964 rs9908511 rs1075009	Obesity, smoking	
		11 *OR52K1* rs96489 rs331508 rs331510 rs4468345	Obesity	
		1 *OR2L8* rs4925583 rs4925792		Obesity
		10 *CALML3* rs1131482 rs2231413 rs1142825 rs4072071 rs4072070 rs4589188 rs4589189	Smoking	Obesity

VAT—visceral adipose tissue, BF—body fat, TAT—total adipose tissue, SAT—subcutaneous adipose tissue area (measurements were provided using underwater weighing and computed tomography), WC—waist circumference, BMI—body mass index, BW—body weight.

**Table 2:** Overview of Olfactory Receptor Gene Variants. This table presents the percentages of specific pseudogene alleles in various olfactory receptors, the odors detectable by these receptors, and their corresponding natural agonists. The data elucidates the genetic diversity of olfactory receptors and their roles in odor perception.

Study and Number of Participants (N)	Method	Odorant Receptor Name	Pseudogene Allele Percentage	Resulting Odor to Be Detected	Natural Agonist
Mainland [75] 2013 N = 511	For sequencing, human genomic DNA was amplified with HotStar Taq (Qiagen)	*OR2B11*	43%	8-amino-acid protein	Cinnamaldehyde
		*OR4E2*	30%	MAYDRY domain	Amyl acetate
		*OR8K3*	24%	MAYDRY domain	(+)-menthol
		*OR10A6*	22%	PMLNPLIY domain	3-phenyl propyl propionate
		*OR2C1*	4%	272 amino acid protein	Octanethiol
		*OR4Q3*	1.5%	159 amino acid protein	Eugenol
		*OR10G7*	1.4%	191 amino acid protein	Eugenol
		*OR10G4*		guaiacol, vanillin and ethyl vanillin	

**Table 3:** Comprehensive Overview of Multiple Studies on Olfactory Receptor Gene Variants and related Odor Perception: Correlating Genetic SNPs with Sensitivity for Specific Odors.

Study and Number of Participants (N)	Method	Chromosome Odorant Receptor Variants (SNPs)	Related Odors to Be Detected
Eriksson [79] 2009 N = 22 studies	Genotyped on the Illumina HumanHap550+ BeadChip platform	1 *OR2M7* rs4481887 rs4309013 rs4244187	Smell of asparagus metabolites in urine
Eriksson [80] 2012 N = 26691	Genotyping by Beagle and Minimac	11 *OR6A2* rs72921001	Aldehydes (cilantro)
Jaegaer [81] 2009 N = 48	Microarray probe genotyping	6 *OR2W1*	Alcohols including 1-hexanol
		6 *OR2J2*	
		6 *OR2J3* rs28757581	Cis-3-hexen-1-ol (fruits, vegetables, white wine and processed foods)
		6 *OR2J3* rs3749977	
Lunde [74] 2012 N = 23	For sequencing, human genomic DNA was amplified with HotStar Taq (Qiagen)	*OR7D4* rs61729907	Androstenone (cooked pork)
		*OR7D4* rs5020278	
Menashe [82] 2007 N = 377	Matrix-assisted laser desorption/ionization–time of flight (MALDI-TOF	*OR11H7*	Isovaleric acid (sweaty odor)

### 3.2. Olfactory Sensory Perception and Metabolism

We found eight original studies reporting a connection between olfactory function and obesity. In a study by Massol et al., it was found that the odor of dark chocolate reduced the appetite and ghrelin levels in young women [83]. The study by Ketterer et al. added to our understanding of the metabolic influences on olfactory function by examining the effects of systemic insulin levels on olfactory thresholds in healthy individuals. Utilizing a hyperinsulinemic–euglycemic clamp approach, the study demonstrated an increase in the olfactory threshold (from 7.8 ± 1.2 to 6.2 ± 1.1, *p* = 0.0173) during hyperinsulinemia, while no significant change was noted in the fasting control group. These findings highlight the potential of insulin to adjust olfactory sensory perception in the postprandial state, characterized by a reduction in food seeking [84]. It is of note that insulin resistance is associated with impaired olfactory identification, although this interaction was not notably linked to the BMI and HOMA index [85]. To the contrary, the administration of intranasal insulin to patients with post-infection loss-of-smell improved olfactory sensitivity. Additionally, patients with a higher BMI also showed improvements in odor identification tasks upon intranasal insulin administration [86]. Another study indicated that the presence of insulin in the olfactory bulb could be connected with the process of satiation and the pathogenesis of obesity [72]. GLP-1 agonist treatment decreases the olfactory preference for sweet- and fat-enriched food [87] and improves olfactory sensitivity and odor-induced right parahippocampal activation [88]. In both cases, it remains unclear whether the observed effects were directly linked to GLP-1 analogue effects on the olfactory system or indirectly resulted from the reduction in body weight. 

The interventional impairment of olfactory sensory perception using a specific intranasal device resulted in a reduction in body weight, improved insulin sensitivity, and decreased preferences for sweet foods in subjects aged ≤ 50 years with obesity on a low-calorie diet compared to a control group. Interestingly, no effect was observed in study participants > 50 years of age [89]. As olfactory function seems to deteriorate with aging [90],^,^ this might explain why interventions to reduce olfactory sensory perception are not effective at an advanced age.

### 3.3. Olfactory Function and Obesity 

We found 11 original studies reporting alterations in olfactory function in individuals with obesity. In a study by Velluzzi et al., only 34% of participants with overweight were normosmic, as opposed to 66% of healthy participants. When the overweight group was further divided into participants with overweight and obesitye, the authors observed that there was a negative correlation between the BMI and olfactory function [91]. In another population-based study, the data were similar, reporting hyposmia in 34.9% and anosmia in 31.7% of individuals with overweight and hyposmia in 34.7% and anosmia in 27.5% of individuals with obesity. It is important to note that the mean age in this study was 71.1 years, which could have influenced the result [92]. Campolo et al. further reported that, in subjects with obesity, olfactory impairment is highly common and associated with poor sleep quality and a lower cognition score [93]. Other studies showed that an increase in the BMI and visceral fat are linked to declining odor perception and olfactory capacity [24,31,77,94,95]. Rawal et al. also observed that self-reported olfactory dysfunction is positively correlated with BMI. Subjects with olfactory dysfunction were observed to have a BMI of 30.0 ± 0.3, while the normosmic group had a BMI of 29.2 ± 0.2 (*p* < 0.05). They also observed that the normosmic group consumed fewer calories per day, especially less additional sugar, and showed a healthier food preference than the hyposmic group [96]. A study by Patel et al. suggested that an increasing BMI may be a risk factor for anosmia and limited control of food consumption [97]. In a study examining the influence of odors on children’s food choices, 45 normal-weight and 29 obese children were enrolled. The study used pear and pound cake aromas as olfactory primes, selected for their appeal and recognition by children. The key finding was that the impact of these aromas on food choice differed according to their body weight. In normal-weight children, both pear and pound cake odors significantly decreased the likelihood of choosing fruit compared to the control condition. Conversely, in obese children, the pear odor increased the probability of selecting fruit, while the pound cake odor had no significant effect on their food choices. This differential response underscores that the non-conscious perception of specific olfactory cues—fruity (pear) and fatty-sweet (pound cake)—can influence food choice differently in children depending on their body weight status [98].

### 3.4. Olfactory Function and Bariatric Surgery

Following the inclusion of 51 articles, we selected 14 studies for the meta-analysis due to their comparable quantitative data. These studies examined the relationship between bariatric surgery and olfactory function. The characteristics of the included studies are presented in Table 4. 

In total, the studies encompassed data from 1402 participants. Among them, 724 underwent Roux-en-Y gastric bypass (RYGB), 537 received sleeve gastrectomy (SG), and 34 underwent another type of bariatric surgery. The median follow-up period was six months. Of the 14 studies, 5 either lacked a control group or did not specify the type of surgery. Different tools were applied to assess olfactory function. Seven studies used the Sniffin’ Sticks test combined with the threshold, discrimination, and identification (TDI) score; one used the Pocket Smell test (PCT); five employed a visual analogue scale (VAS); and one utilized the Cross-Cultural Smell Identification test (CC-SIT). 

**Figure 3:**
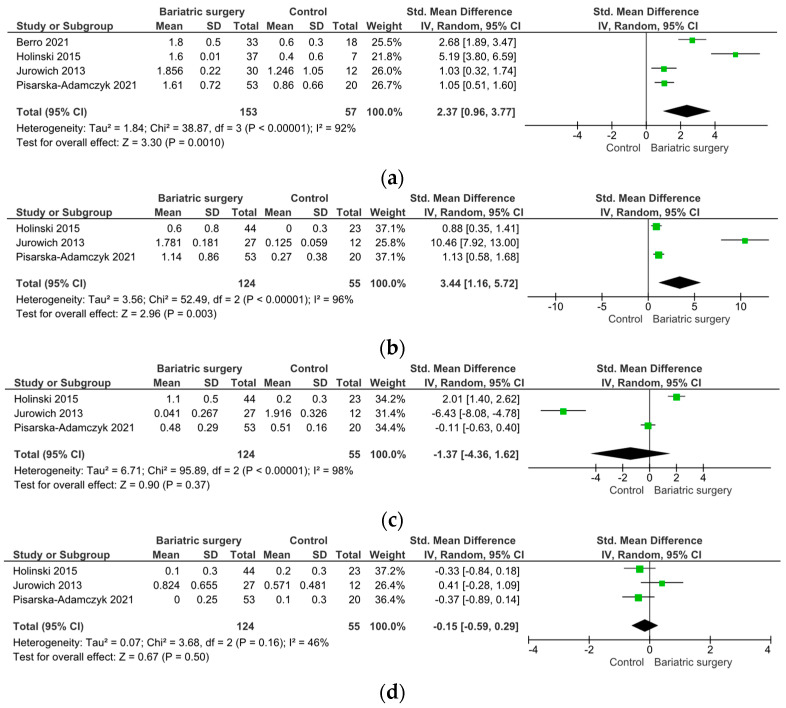
Changes in olfactory function parameters upon bariatric surgery versus control group. (**a**) Total TDI, (**b**) threshold, (**c**) discrimination, and (**d**) identification.

Four studies indicated improvements in OF post-bariatric surgery, assessed via the total TDI score using the Sniffin’ Sticks test (SMD 2.37, 95% CI [0.96, 3.77], I = 92%, *p* = 0.001) (Figure 3a) [99,104,105,109]. There was a significant improvement in the olfactory threshold post-bariatric surgery (SMD 3.44, 95% CI [1.16, 5.72], I = 96%, *p* = 0.003) (Figure 3b), while olfactory discrimination and identification remained unaffected (*p* > 0.05) (Figure 3c,d). 

**Figure 4:**
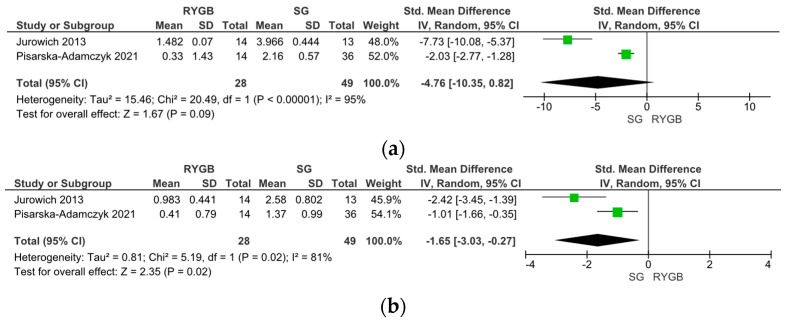

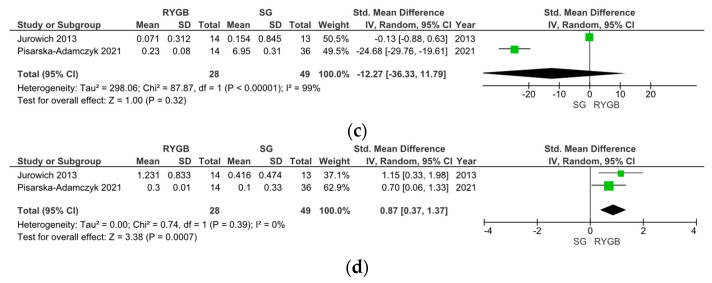
Comparison of Roux-en-Y Gastric Bypass (RYGB) versus Sleeve Gastrectomy (SG). Changes in olfactory function according to olfactory identification (TDI).

Five studies compared the changes in olfactory function between RYGB and SG surgery. In two studies, olfactory function was assessed using the Sniffin’ Sticks test with TDI scoring, while three studies used the VAS score; therefore, the data were analyzed separately. Notably, the Sniffin’ Sticks test with TDI demonstrated a significantly stronger improvement in OF post-SG than post-RYGB surgery (SMD −4.76, 95% CI [−10.35, −0.82], I = 95%, *p* = 0.09) (Figure 4a). Upon SG, an improvement in the olfactory threshold (SMD −1.65, 95% CI [−3.03, −0.27], I = 81%, *p* = 0.02) (Figure 4b), no changes in olfactory discrimination (SMD −12.27, 95% CI [−36.33, −11.79], I = 99%, *p* = 0.32) (Figure 4c), and a decrease in olfactory identification (SMD 0.87, 95% CI [0.37, 0.33], I = 0%, *p* < 0.001) (Figure 4d) were observed. 

**Figure 5:**
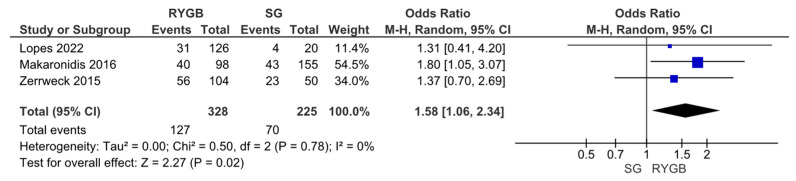
Changes in olfactory function according to visual analogue scale (VAS) score upon Roux-en-Y Gastric Bypass (RYGB) versus Sleeve Gastrectomy (SG).

However, an assessment via VAS scores revealed the opposite outcome, showing a stronger improvement of olfactory function parameters upon RYGB compared to SG (OR 1.58, 95% CI [1.06, 2.34], I = 0%, *p* = 0.02) (Figure 5).

Four studies without a control group demonstrated an improvement in the total TDI score upon bariatric surgery [100,103,108,112]. Two other studies also lacking a control group showed an improvement in olfactory function following bariatric surgery, as assessed using VAS scores in combination with a questionnaire [101,102]. Only one study compared olfactory performance upon RYGB with a control group using VAS scores, showing a superiority of RYGB over the control group regarding the improvement of olfactory performance (OR 1.22, 95% CI [0.11, 14.09], *p* = 0.87) [110].

**Figure 6:**
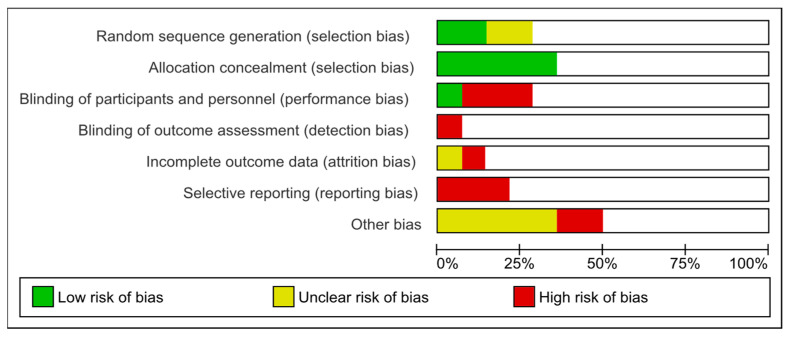
Risk of bias of the studies investigated.

Figure 6 provides an overview of risk of bias of the studies investigated.

## 4. Discussion

In this systematic review and meta-analysis, we observed a correlation between increased BMI and changes in olfactory function. This association was characterized by a higher incidence of anosmia and hyposmia among individuals with overweight and obesity. However, it is important to note that these findings indicate a relationship, not a causal link. Additionally, individuals with overweight, but not obesity, who developed hyposmia reported a reduced food consumption and a lower preference for high-calorie foods [113]. 

For decades it was believed that humans can distinguish up to 10,000 different odors. However, in 2014 Bushdid et al. challenged this view by testing and calculating the capacity of humans to discriminate odor mixtures, revealing that at least one trillion different odors can be recognized by humans [114], although the results were debated [115]. While humans have around 400 different olfactory receptors (ORs), mice have more than 1000 ORs [116]. OR genes represent some of the largest gene families among vertebrates [117] and code G-protein-coupled receptors (GPCRs), each of which can detect different odor molecules [20,21]. Copy number variations, which are common in OR genes, lead to a high diversity in odor perception capability [22]. Variations in distinct ORs genes are associated with obesity, BMI, waist circumference, and daily calorie intake. Although the direct link and underlying mechanisms of genetic variations in OR genes in regard to metabolism regulation remains to be demonstrated, these findings indicate a link between olfactory performance and systemic energy homeostasis, and possibly eating behavior in humans [118]. Notably, ORs are not only expressed by olfactory sensory neurons, but by almost every tissue in the rodent and human organism, whereby the relevance is unclear [119]. At the level of intestinal enterocytes, ORs may direct food choice depending on the fat content of the food [120]. 

Other studies have shown that temporary alterations in the general health condition of individuals, including nasal infections or allergic rhinitis, may have concomitant, yet temporary, effects on olfactory function and subsequently on systemic metabolism [121,122,123]. On the other hand, changes in systemic metabolism impact olfactory function. In this review and meta-analysis, we found that bariatric surgery has a positive effect on olfactory function. During a follow-up period of at least 6 months, an improvement in smell and olfactory sensitivity was detected in ten studies assessing olfactory performance via a Sniffin’ Sticks test combined with TDI scoring or VAS. One study showed that while participants with obesity had a decreased olfactory performance, as assessed via Sniffin Sticks test and TDI scoring, participants with anorexia had an improved overall olfactory function [26]. The latter finding was contradicted in the study by Enck et al., although the normal weight control group was not age-matched in their study [100].

A separate analysis of the odor threshold, discrimination, and identification using the TDI score revealed that the distinct dimensions of olfactory sensory perception are differentially impacted upon bariatric surgery. The odor threshold was significantly improved after bariatric surgery, especially upon SG. These findings build upon previous studies reporting a lowered odor threshold with increasing body weight [31,91,124,125], while olfactory discrimination and identification does not seem to be impacted by body weight or obesity in humans [25,33,126], although these findings are still a subject of debate [91]. This impairment in the olfactory threshold might be mediated via proinflammatory cytokines or leptin [95,126]. In contrast to the olfactory threshold, olfactory discrimination and identification did not change upon bariatric surgery when compared to control groups. However, comparing RYGB and SG surgery, olfactory discrimination and identification scores were significantly better upon RYGB compared to SG.

The majority of studies used the widely accepted and validated ‘Sniffin’ Sticks’ test battery [127] combined with TDI scoring. In addition, there is a wide variety of unstandardized tests to determine olfactory function, such as the Smell Identification Test (UP-SIT), which is based on the identification of scents on each page of four booklets [128], the Pocket Smell Test (PST), which is based on the ability to determine the correct smell from the proposed options [129], and the CC-SIT, which is based on the same principles as the previous two tests with adaptions to a specific cultural background usually consisting of 12 questions [130]. Moreover, olfactory function is widely assessed via VAS, which is based on a person’s subjective self-assessment [131] and thus is prone to bias. Interestingly, olfactory function, as assessed via the Sniffin’ sticks test, seems to improve after bariatric surgery, specifically after SG [99,100,103], but not after RYGB [110]. This might be explained by the stimulation of the vagus–insula–olfactory cortex pathway which is suppressed in obesity and restored upon SG, but not upon RYGB, despite comparable weight loss [104,105,106,107,108,111]. 

It is interesting and somewhat puzzling that olfactory performance is significantly more improved upon RYGB compared to SG when assessed via VAS. VAS is validated as a reliable method for the assessment of several health conditions [132,133]. However, changes in olfactory performance might be too small to be correctly captured via VAS. Moreover, VAS is not able to assess the different dimensions of olfactory sensory perception. Therefore, olfactory function outcomes based on VAS should be interpreted with caution. 

The studies incorporated in this systematic review present several limitations. Firstly, five of the studies lacked control groups. Additionally, all of the included studies were non-randomized and often involved only a small cohort of participants. A notable concern was the absence of standardized methods to evaluate olfactory function, which complicated direct comparisons between studies. Although many studies employed the TDI test—considered by many as the objective gold standard—others utilized the VAS score. The latter introduces a greater potential for bias and poses challenges when attempting to compare results across individuals and distinct therapeutic procedures.

## 5. Conclusions

In summary, our review and meta-analysis revealed that variants in OR genes are associated with metabolic changes at the systemic level in humans where underlying mechanisms are elusive. Moreover, we found that olfactory performance is negatively correlated with BMI. Our findings suggest that the most prevalent bariatric surgery procedures, RYBG and SG, result in an improved olfactory sensory perception, particularly regarding the olfactory threshold, among patients with obesity. When assessed via the VAS score, RYBG led to a more pronounced improvement in olfactory function compared to SG. However, when evaluated with the Sniffin’ Sticks test, SG resulted in a significantly improved olfactory performance compared to RYGB.

## Figures and Tables

**Table 4:** Characteristics of the 14 publications investigating the impact of bariatric surgery on olfactory function included in the meta-analysis. Sleeve Gastrectomy (SG), Roux-en-Y gastric bypass (RYGB), mini gastric bypass (mGB), adjustable gastric band (AGB) Pocket Smell test (PST), threshold discrimination identification (TDI) test, visual analogue scale (VAS), Cross-Cultural Smell Identification test (CC-SIT).

Study, Year Number of Participants (N) Age (Mean) BMI (kg/m^2^)	Design	Type of Surgery	Type of Olfactory Function Assessment	Follow-Up (Months)	Bariatric Surgery Group (N, BMI)	Control Group (N, BMI)
Berro, 2021 [99] N = 34 Age = 46.4 y.o.	Prospective longitudinal study	LSG	TDI	6	N = 18 BMI = 42.6 kg/m^2^	N = 16 BMI = 43.1 kg/m^2^
Enck, 2014 [100] N = 8 Age = 47.6 y.o.	Repeated measures design	SG	TDI	12	N = 8 Age = 47.6 y.o.	-
Graham, 2014 [101] N = 103 Age = 45 y.o.	Retrospective cohort study	RYGB	VAS	12 & ≥36	N = 103 BMI = 51 [36–97] kg/m^2^	-
Guyot, 2021 [102] N = 220 Age = 41.0 y.o. BMI = 42.3 kg/m^2^	Cross-sectional Study	RYGB, 56% had an SG, and 12% had other procedures Omega-loop gastric bypass N = 16 Gastric band N = 6 Fundoplication N = 2 Banded gastric bypass N = 1 Biliopancreatic derivation with duodenal Switch N = 1 Single anastomosis duodenum–ileal bypass (N = 1)	VAS	24	N = 220 BMI = 42.3 [30.4–64.6] kg/m^2^	
Hanci, 2015 [103] N = 54 Age = 37.1 y.o. BMI 44.8 kg/m^2^	Prospective cohort design	SG	TDI	6	N = 54 BMI 44.8 kg/m^2^ [30.5–63.0] kg/m^2^	-
Holinski, 2015 [104] N = 67 Age = 47.1 y.o.	Prospective cohort study	AGB, SG, RYGB	TDI	6	N = 44 BMI = 48.6 kg/m^2^	N = 23 BMI = 23.4 kg/m^2^
Jurowich, 2013 [105] N = 42 Age = 43.4 y.o. BMI = 51.70 ± 7.36 kg/m^2^	Prospective case–control observational study	RYGB, SG	TDI	6	RYGB N = 15 BMI = 48.7 ± 5.3 kg/m^2^ SG = 15 BMI = 56.0 ± 6.2 kg/m^2^	N = 12 BMI = 49.49 ± 4.73 kg/m^2^
Lopes, 2022 [106] N = 151 Age = 42 y.o. BMI = 32.6 km/m^2^	Prospective observational study	RYGB, SG	VAS	12	RYGB N = 126 BMI = 32.7 kg/m^2^ SG N = 20 BMI = 32.0 kg/m^2^	
Makaronidis, 2016 [107] N = 253 Age = 45.4 y.o.	Prospective observational study	RYGB, SG	VAS	6–60	RYGB N = 98 BMI = 44.7 kg/m^2^ SG N = 155 BMI = 46.1 kg/m^2^	-
Melis, 2021 [108] N = 51 Age = 44.6 y.o. BMI = 43.0 kg/m^2^	Prospective cohort study.	RYGB, SG, mGB	TDI	6	N = 51 BMI = 43.0 kg/m^2^	-
Pisarska-Adamczyk, 2011 [109] N = 88 Age = 46.6 y.o.	Prospective study	RYGB, SG	TDI	6	N = 53 BMI = 45.4 kg/m^2^	N = 35 BMI = 24.9 kg/m^2^
Richardson, 2011 [110] N = 95 Age = 40.5 y.o.	Prospective study	RYGB + prophylactic cholecystectomy	CC-SIT	6–12	N = 55	N = 40 (cholecystectomy)
Zerrweck, 2015 [111] N = 154 Age = 41.2 y.o.	Cohort study	RYGB, SG	VAS	10	LGBP N = 104 BMI = 43.3 kg/m^2^ LSG N = 50 BMI = 45.8 kg/m^2^	-
Zerrweck2017 [112] N = 59 Age = 47.1 y.o. BMI = 46.9 km/m^2^	Prospective cohort study	RYGB	PCT	6	N = 30	N = 29 (nasofibroscopy)

## Data Availability

The data supporting the findings of this study were derived from previously published articles and are available in the public domain. Details of the specific studies included in this systematic review and meta-analysis can be found in the referenced articles and within the PROSPERO registration (CRD42022355091). No new data were created or analyzed in this study.
